# Lifetime Health Care Costs of the Danish Population

**Published:** 2013-10-09

**Authors:** Jan Sørensen, Rikke Søgaard

**Affiliations:** 1 Centre for Applied Health Services Research University of Southern Denmark, Odense, Denmark

**Keywords:** general population, lifetime cost, denmark

## Abstract

**Objectives:** Lifetime health care cost is a statistical indicator for expected health care use during the remaining lifetime of an individual. The objective of this study was to estimate the lifetime health care costs for individual gender and age strata.

**Methods:** Register data on individual’s health service utilization (hospital services, primary care and prescription medication) were available for all Danes above age 16 years (N=4.3 millions) for the year 2006. Resource use was valued by national diagnosis-related group (DRG) tariffs and fees. Average yearly costs were derived for gender and age stratas. The life table method was used to estimate survival probabilities and expected lifetime health care costs.

**Results:** The average lifetime health care cost for a 16-year-old person was estimated at 693,000 Danish Kroner (DKK) (2006 values) for men (216,000 DKK when discounted by 3%) and 862,000 DKK for women (301,000 DKK when discounted by 3%). Substantial variation was observed across gender and age strata with a relatively higher load late in life. Lifetime health care costs were higher for women than men. Part of the gender difference relates to longer life expectancies (estimated at 79.5 for women and 75.1 years for men). The analysis suggests that longevity of life accounts for 78% of the difference in health care costs between men and women.

**Conclusion:** This study presents cost data for the general population that can be used to inform general economic models assessing the impact of aging populations, but also comparative models that seek to assess the efficiency and cost-effectiveness of new technologies. Future studies that address similar indicators for disease-specific populations will be valuable.

## 1. Introduction

The cost of health care is an important aspect for the assessment of efficiency and cost-effectiveness of health technologies. The incremental cost associated with a new technology is usually compared with the incremental effect in the so called incremental cost-effectiveness ratios (ICER). Such comparisons should be based on an analytical time perspective, which is sufficiently long to capture all differences between the technologies analyzed, which often means a lifetime perspective.[1] In practice, however, it is rarely feasible to observe costs for the remaining lifetime of a study population.

[1]Centre for Applied Health Services Research, University of Southern Denmark, Odense, Denmark

Investigators therefore often turn to models that estimate long-term costs based on observations for a shorter period of time. Two different approaches can be applied. The first is based on longitudinal data with repeated measurements for a defined cohort of individuals. The advantage of this approach is that the between-person heterogeneity can be controlled for. On the other hand, the effects of age can confound the effects of time, e.g. due to technological advancements or price deviations.[2-4] The second approach is based on cross-sectional data where different birth cohorts are used to represent different age strata. The advantage of this approach is that the effect of time is controlled for, whereas the between-birth-cohort heterogeneity may lead to confounding.[5]

In order to establish reference values for age- and gender-specific strata of a population it should be clear that, yet infeasible, the optimal approach would be a difference-in-difference design, i.e., observing differences over time for different birth cohorts. One disadvantage of these models are that they are sensitive to the functional forms of the models. Models that use a log-linear approach would provide different results than models using ordinary least squares. Further, a trade-off must be made between the possible confounding from the course of time (using the longitudinal design) and the confounding from characteristics of different birth cohorts (using the cross sectional design). In this paper we develop a model, which is based on the cross sectional approach but which additionally includes the different life expectancies of different birth cohorts. A similar although not identical approach has been previously demonstrated by Alemaeyhu and Warner.[6]

To the best of our knowledge, there exist no reference values for the lifetime health care costs of the Danish general population. Several cost-of-illness studies have estimated lifetime costs for various diseases, but the methodology is typically based on projections of relatively short time periods observed.[7,8] At the same time, the availability and quality of national register data have increased over recent years, which makes it possible to base empirical studies on observations for the entire population.[9]

The objective of this study was to estimate lifetime health care costs for individual gender and age strata for the Danish, adult population.

## 2. Methods

The study takes advantage of individual person-level register data for health care use and mortality during the year 2006 for the entire adult population. These data are used to observe the annual health care costs for different age and gender strata and to construct a hypothetical lifetime cost distribution based on the life table method. The following sections outline the analytical approach and the specific data used.

### *The Life Table Method*

To estimate lifetime health care costs we used the method known as the period life table model method. We use a single year's average health care cost and mortality rates, observed for specific age- and gender strata, to generate profiles of total health care costs for hypothetical individuals from 16 years of age to death. This framework assumes steady state, which explicitly means that health technology and associated costs, the prevalence, incidence and natural history of diseases remain unchanged at the current level throughout the lifetime of the modeled individuals.

A period life table starts with a cohort of 100,000 hypothetical individuals to whom observed age- and gender-specific mortality rates are applied. The life table shows the mortality experience of a hypothetical group of individuals born at the same time and throughout their lifetime subject to the age-specific death rates observed in the current period. For each age and gender strata, the average cost of health care is calculated separately for individuals surviving the year and those who died during the year. These cost figures are multiplied with the number of individuals in the gender and age strata. By accumulating the costs of individuals during the lifetime and dividing these with the number in the initial cohort (100,000 individuals), the expected lifetime cost is derrived.

The expected lifetime cost (*LC*) for an individual of age *a* can thus be defined as:

(1) $$LC^a = \sum_{t=a}^{T}\left[s_t\, c_t^{surv} + (1 - s_t)\, c_t^{die}\right]$$

where *t* denotes age and *T* denotes the analytical truncation age; $s_t$ denotes the survival probability until *t+1* for individuals alive at the age *t*; $c_t^{survive}$ and $c_t^{die}$ denote the annual cost rate for individuals at age *t* who either survive the full year or die during the year.

In the present analysis our time unit is calendar years and age is truncated at *T*=100 years. No discounting was applied in the base-case analysis, but various annual discount rates *r* were used in alternative analyses (1.5%, 3% and 5%) according to the conventional formula:

(2) $$LC^a = \sum_{t=a}^{T}\left[s_t\, c_t^{surv}(1 - r)^{-t} + (1 - s_t)\, c_t^{die}(1 - r)^{-t}\right]$$

We stratify $s_t$, $c_t^{survive}$ and $c_t^{die}$ for men and women and report the lifetime cost $LC^a$ as the average within 10-year age groups. Furthermore, the lifetime cost $LC^a$ is split according to the type of health care: general practice, private medical specialists, physiotherapists, dentists, other primary care providers, pharmaceuticals, outpatient and inpatient hospital contacts.

In order to validate the data preparation and analytical approach we estimate overall life expectancies at birth for comparison with national statistics. As individual data were not available for the younger age groups, we used aggregated national statistics for the age group 0-15 years.

Data

We obtained data from the national civil registry,[10] the cause of death registry,[11] the national patient registry,[12] the primary care registry[13] and the primary care prescription registry,[14] all relating to the year 2006.

The civil registry holds information about all residents and their unique identification number (here we used a scrambled civil registration number to identify individuals). We obtained identification of all individuals aged 16 years or older who were alive by January 1, 2006. The variables obtained included date of birth and gender.

From the registry of cause of death we obtained date of death for individuals deceased during 2006.

From the national patient registry we obtained data on all inpatient and outpatient contacts during 2006. The variables included type of admission and the associated cost data from the case-mix system of Diagnosis-related grouping (DRG) for inpatient services and Danish Outpatient Grouping System (DAGS) for outpatient visits.[15]

From the primary care registry we obtained weekly aggregated data on number of services provided by different service providers and total fees paid to the service providers. We categorized service providers into five groups: (general practice (code 80-84), medical specialists (code 1-41), therapists (code 51-65, 68), dentists (code 50) and other providers (all other codes).

Finally, from the pharmaceutical registry we obtained data for all prescription medicine provided by pharmacies in the primary sector and the total price paid to the pharmacy by patients, regions and municipalities.

All cost data are reported in local currency (Danish Kroner [DKK]) at 2006-price level. To adjust the cost to 2013-price level the net price index reported by Statistics Denmark may be used (mid-year net price index 2006: 113.7; mid-year 2013: 131.4). To adjust local currency rates to EURO or US$ the mid-year exchange rates obtained from the Organisation for Economic Co-operation and Development (OECD) StatsExtracts system may be used (2006: 1 DKK = 0.127 EURO = 0.171 US$) and (2013: 1 DKK = 0.131 = 0.175 US$).

## 3. Results

The entire Danish population over age 16 years amounted to 4.3 million individuals at the beginning of year 2006. The gender and age distribution is illustrated in Figure 1. The 2006 age-specific death rates per 1,000 men and women are shown in Figure 2. The average annual health care costs for different aged men and women who survived or died during 2006 are shown in Figure 3.

**Figure 1. Age Distribution of the Danish Population Alive on 1 January 2006 (age 16 or more)**

**Figure 2. Annual Death Rates per 1,000 at Risk (in 2006)**

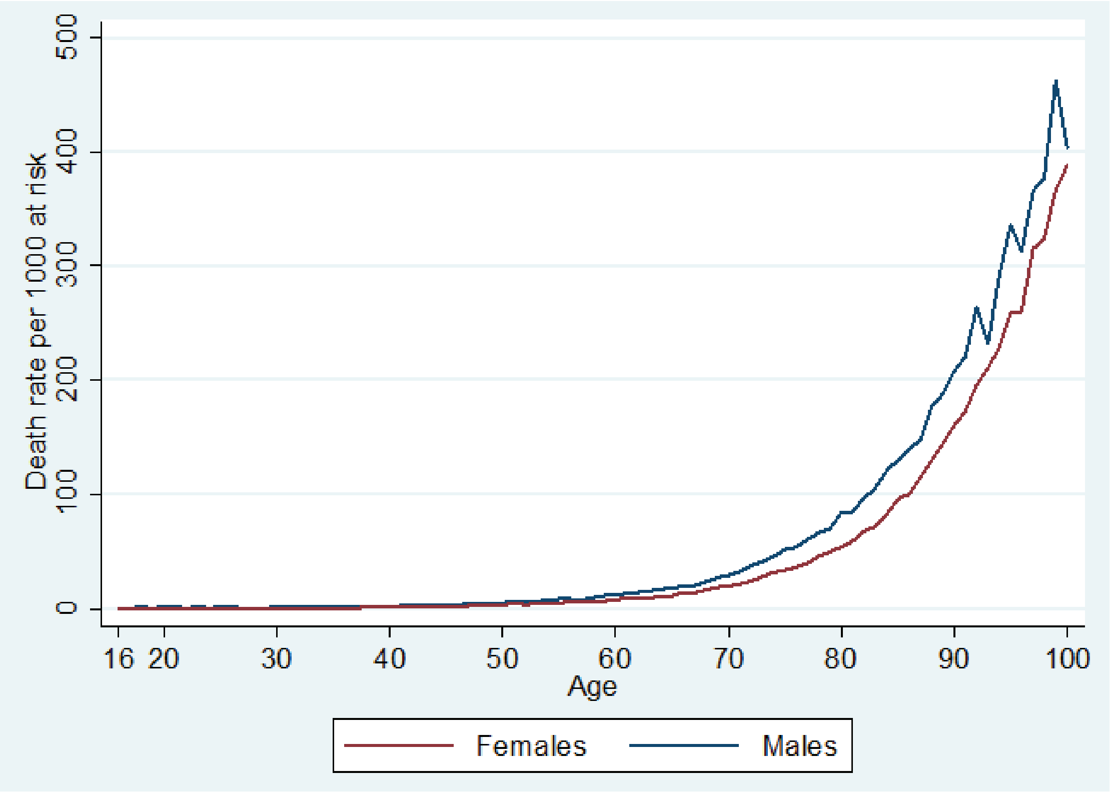

**Figure 3. Average Health Care Costs for Men and Women who Survivied or Died during 2006 (2006-DKK)**

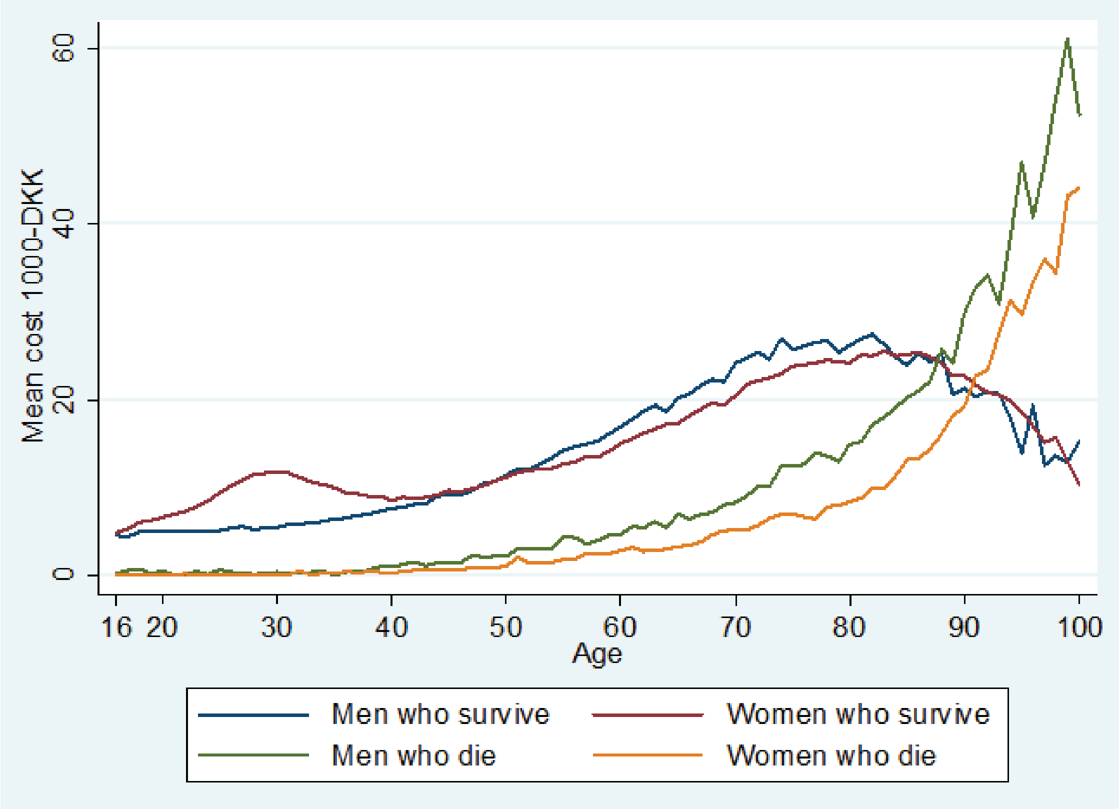

Note: 1 DKK = 0.127 EURO = 0.171 US$ (2006 prices)

Assuming all exogeneous parameters to be constant over time, lifetime health care costs of the Danish population are illustrated in Figure 4. As expected, the overall rates of costs increase from early to the later years of life, except that women demonstrated a minor increase in their years of fertility. The average annual costs for men aged 16-24 was 3,800 DKK, while costs for men aged 75-84 were 8.5 times higher, at 32,200 DKK; a similar factor applies to women. Having peaked in the 80s for both genders, those alive toward the century mark demonstrated declining costs.

**Figure 4. Average Health Care Costs per Person 2006 (2006-DKK)**

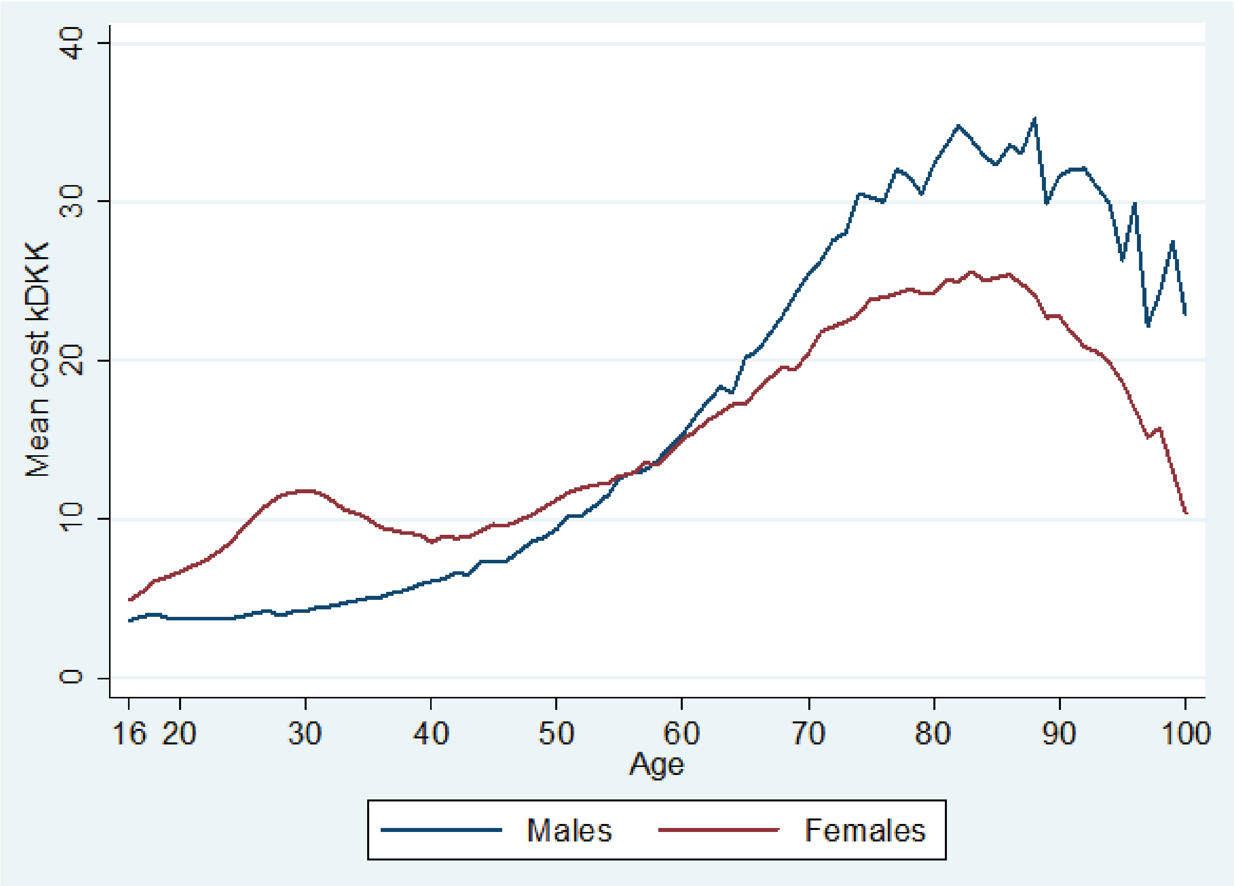

Note: 1 DKK = 0.127 EURO = 0.171 US$ (2006 prices)

Table 1 details the annual costs across different cost categories. It appears that 71% and 65% of the annual costs relate to hospital use of men and women, respectively, while 17% and 14% relate to pharmaceuticals and 21% and 11% of costs were related to primary care. This distribution is reasonably stable in the different age groups, although a larger proportion of annual costs relates to general practice for the younger ages.

**Table 1. Average Annual Health Care Costs of the Danish General Population Stratified by Gender and Age (2006-DKK)**

| | All Health Care Costs | General Practice | Medical Specialist | Therapist | Dentist | Other Primary Care Provider | Pharmacy | Hospital Outpatient Care | Hospital Inpatient Care |
|---|---|---|---|---|---|---|---|---|---|
| **Men** | | | | | | | | | |
| 16-24 | 3,752 | 373 | 168 | 76 | 148 | 24 | 517 | 910 | 1,534 |
| 25-34 | 4,274 | 400 | 223 | 107 | 211 | 38 | 816 | 897 | 1,580 |
| 35-44 | 5,942 | 475 | 265 | 139 | 265 | 37 | 1,186 | 1,141 | 2,435 |
| 45-54 | 9,220 | 593 | 320 | 174 | 336 | 41 | 1,889 | 1,565 | 4,303 |
| 55-64 | 15,225 | 793 | 422 | 222 | 385 | 52 | 2,935 | 2,539 | 7,877 |
| 65-74 | 24,754 | 1,213 | 581 | 305 | 347 | 68 | 4,419 | 4,059 | 13,761 |
| 75-84 | 32,226 | 1,661 | 680 | 326 | 241 | 90 | 5,570 | 4,131 | 19,528 |
| 85+ | 29,453 | 1,870 | 391 | 205 | 93 | 95 | 4,745 | 2,346 | 19,708 |
| All Men | 16,722 | 996 | 384 | 196 | 243 | 59 | 2,926 | 2,224 | 9,694 |
| **Women** | | | | | | | | | |
| 16-24 | 6,218 | 856 | 317 | 131 | 184 | 70 | 1,024 | 1,323 | 2,313 |
| 25-34 | 10,390 | 1,040 | 480 | 195 | 247 | 106 | 1,318 | 2,469 | 4,535 |
| 35-44 | 8,555 | 850 | 520 | 255 | 297 | 72 | 1,647 | 1,745 | 3,167 |
| 45-54 | 10,274 | 853 | 590 | 335 | 373 | 65 | 2,316 | 1,879 | 3,863 |
| 55-64 | 14,262 | 940 | 636 | 357 | 398 | 66 | 3,310 | 2,446 | 6,109 |
| 65-74 | 21,309 | 1,275 | 735 | 378 | 334 | 80 | 4,835 | 3,139 | 10,533 |
| 75-84 | 27,796 | 1,629 | 756 | 351 | 229 | 107 | 6,103 | 3,193 | 15,428 |
| 85+ | 27,256 | 1,694 | 396 | 228 | 79 | 116 | 5,482 | 1,970 | 17,291 |
| All Women | 16,681 | 1,184 | 545 | 277 | 255 | 88 | 3,438 | 2,261 | 8,633 |

Note: 1 DKK = 0.127 EURO = 0.171 US$ (2006 prices)

Table 2 shows the undiscounted lifetime health care cost for different age strata of men and women. An average 16-year-old man is expected to inucr 693,000 DKK in health care costs, while a women of similar age is expected to incur 862,000 DKK. Over their lifetime women thus use 24% more health care resources than men. In a lifetime perspective, more than 68% of the undiscounted lifetime costs for men are related to hospital services, 18% to primary care prescription medicine and 13% to primary care. The similar figures for women - 63%, 21% and 17%, respectively, indicate that women use slightly higher proportions of primary care and pharmaceuticals than men.

**Table 2. Lifetime Cost of Total and Specific Health Care Utilization for Different Age and Gender Groups (2006-DKK)**

| | All health Care Costs | General Practice | Medical Specialist | Therapist | Dentist | Other Primary Care Providers | Pharmacy | Hospital Outpatient Care | Hospital Inpatient Care |
|---|---|---|---|---|---|---|---|---|---|
| **Men** | | | | | | | | | |
| 16 | 692,526 | 41,671 | 20,454 | 10,431 | 16,183 | 2,733 | 127,086 | 113,879 | 360,089 |
| 25 | 662,850 | 38,552 | 19,058 | 9,805 | 14,948 | 2,529 | 123,187 | 106,350 | 348,420 |
| 35 | 625,080 | 34,834 | 16,962 | 8,804 | 12,941 | 2,172 | 115,949 | 98,165 | 335,253 |
| 45 | 577,186 | 30,707 | 14,616 | 7,575 | 10,518 | 1,841 | 106,214 | 88,544 | 317,172 |
| 55 | 514,761 | 26,328 | 12,144 | 6,210 | 7,642 | 1,524 | 92,745 | 77,414 | 290,754 |
| 65 | 421,051 | 21,371 | 9,254 | 4,669 | 4,528 | 1,171 | 73,778 | 60,667 | 245,614 |
| 75 | 286,631 | 15,045 | 5,818 | 2,787 | 2,024 | 801 | 49,155 | 35,227 | 175,772 |
| 85 | 150,590 | 8,623 | 2,499 | 1,207 | 652 | 449 | 24,775 | 14,001 | 98,384 |
| **Women** | | | | | | | | | |
| 16 | 861,783 | 66,303 | 35,620 | 17,797 | 18,611 | 5,130 | 176,967 | 142,680 | 398,675 |
| 25 | 807,259 | 58,706 | 32,829 | 16,651 | 16,986 | 4,508 | 168,046 | 131,005 | 378,528 |
| 35 | 705,941 | 48,491 | 28,132 | 14,751 | 14,573 | 3,461 | 155,426 | 106,716 | 334,392 |
| 45 | 627,708 | 40,473 | 23,209 | 12,350 | 11,743 | 2,773 | 140,585 | 90,333 | 306,242 |
| 55 | 543,580 | 33,100 | 17,953 | 9,339 | 8,325 | 2,205 | 121,599 | 74,147 | 276,913 |
| 65 | 440,204 | 26,044 | 12,807 | 6,381 | 4,852 | 1,695 | 97,225 | 54,795 | 236,404 |
| 75 | 307,662 | 18,015 | 7,605 | 3,646 | 2,216 | 1,204 | 66,449 | 32,611 | 175,918 |
| 85 | 167,853 | 9,809 | 2,977 | 1,610 | 672 | 690 | 34,604 | 13,528 | 103,962 |

Note: 1 DKK = 0.127 EURO = 0.171 US$ (2006 prices)

Costs incurred among different age categories varies considerably. 10% of men's undiscounted lifetime cost arises during age 16-34, 16% during age 35-54, 33% during age 55-74 and 41% for those over age75 years. The similar figures for women are 18%, 19%, 27% and 36%. Women thus have higher health care costs in their younger ages, partly due to childbearing and the higher proportion of primary care that accounts for the larger share of health care use in the younger years of life.

Part of the difference in lifetime costs for men and women relates to the longer life expectancy for women. In the present data this difference was estimated at 4.4 years, as the life expectancy for men and women were estimated at 75.1 years and 79.5 years, respectively. This is slightly lower than the official 2006 figures provided by Statistics Denmark (75.9 and 80.5 years), which may be partly explained by our use of 1 year death data, whereas the Statistics Denmark uses averages over 2 years.

In Table 3, the lifetime cost is estimated with different discount rates. With an annual discount rate of 3%, the net present value of the lifetime cost for an individual aged between 16 and 24 years reduces to approximately one third: 216,000 DKK for men and 301,000 DKK for women.

**Table 3. Net Present Value of Lifetime Health Care Costs using Different Discount Rates (1000 2006-DKK)**

| | Undiscounted | 1.5% | 3% | 5% |
|---|---|---|---|---|
| **Men** | | | | |
| 16-24 | 692.5 | 370.0 | 215.9 | 95.4 |
| 25-34 | 662.8 | 389.1 | 244.0 | 116.3 |
| 35-44 | 625.1 | 408.6 | 280.1 | 150.5 |
| 45-54 | 577.2 | 418.5 | 313.5 | 192.3 |
| 55-64 | 514.8 | 410.5 | 334.1 | 234.0 |
| 65-74 | 421.1 | 364.1 | 318.5 | 251.4 |
| 75-84 | 286.6 | 263.5 | 243.5 | 211.2 |
| 85+ | 150.6 | 144.1 | 138.3 | 128.1 |
| **Women** | | | | |
| 16-24 | 861.8 | 483.8 | 300.6 | 150.3 |
| 25-34 | 807.3 | 494.1 | 328.5 | 180.3 |
| 35-44 | 705.9 | 462.4 | 320.4 | 179.3 |
| 45-54 | 627.7 | 449.0 | 333.4 | 203.5 |
| 55-64 | 543.6 | 424.8 | 340.1 | 232.4 |
| 65-74 | 440.2 | 373.1 | 320.7 | 245.8 |
| 75-84 | 307.7 | 278.5 | 253.8 | 214.9 |
| 85+ | 167.9 | 159.4 | 151.8 | 138.8 |

Note: 1 DKK = 0.127 EURO = 0.171 US$ (2006 prices)

## 4. Discussion

This study used population data and a life table model to estimate lifetime health care costs for different age and gender strata of the Danish population. The resulting estimates can be seen as general population reference values that can be used to inform economic models assessing the impact of aging populations and comparative models, such as economic evaluations that seek to assess the efficiency of new technologies.

Our analysis shows the distribution of costs throughout the course of life. It is clear that average annual health care costs are increasing with higher age, until a few years after 80 years. This could indicate that more intensive/costly services are provided to individuals below age 80 years. It could also reflect the disease pattern over the course of life. Younger individuals may suffer from certain types of chronic diseases (e.g. asthma) or those where treatment is costly (e.g. cancer, multitrauma), while individuals who have survived to age 80 may suffer from other diseases that are perhaps are less expensive to treat.

We demonstrated that women use significantly more health care than men over a lifetime. By imposing the women's mortality rates for men, it was possible to assess the impact of length of life on lifetime health care costs. The analysis suggests that women's longer life expectancy accounts for 78% of the undiscounted difference in lifetime costs between men and women. Further, the analysis suggests that if men had the same life expectancy as women, those age 16 years would incurr only 6.5% more health care costs than men.

The results are presented without precision estimates. As the entire Danish general population was included in the study, there is no sampling uncertainty of the estimates and conventional confidence intervals are therefore irrelevant. There is however uncertainty related to the methodological approach of constructing a hypothetical cohort with lifetime follow-up from different birth cohorts. Researchers using the present reference values for average annual health care costs should judge whether their population is comparable to the setting for this study in 2006, in a conventional assessment of external validity.

An intended application for the present results is to provide reference values for subpopulations. A recent study estimated lifetime health care costs for individuals with cerebral palsy at 496,000 DKK for men and 489,000 DKK for women (with a discount rate of 5%).[16] These figures could be compared with those of the general population which, with a 5% discount rate, amounts to 120,000 DKK for men and 183,000 DKK for women, respectively (from age 16). These figures would suggest that individuals with cerebral palsy, on average, use 2.5-4 times more health care resources than the general population. Such odds ratios could be useful in decision analytic models to inform the consequence of preventing cerebral palsy.

Another application of our results may be to project potential consequences of future demographic changes. It has been established with a high degree of certainty, that the population in many European countries will age during the next 10-20 years.[17] Demographic projections indicate that the elderly group especially will expand, and the population group at age 85+ will double in the years to come. By applying our cost estimates to population assumptions, contributions can be made to the ongoing policy and planning debate.

The net present value of the lifetime health care costs decrease with higher discount rates. However, it is clear from the analysis that the timing of health care costs is different for among age strata. Therefore, using a 3% discount rate, the lifetime cost of a patient age 16 is lower than that of one age 55 years. The intuitive explanation is that a 55-year-old person will incur higher health care costs sooner than a 16-year-old person. It is interesting to note that the highest lifetime costs occur for different age groups, depending on the discount rate applied. With a low discount rate, the value of future cost is not as reduced as with a high discount rate.

*Limitations and Future Perspectives*

The main limitation of the study relates to the definition of the cost parameter. We only considered costs of health care services that are registered in the national administrative databases. This means that we have disregarded care of a more social nature, which is provided by the municipalities in Denmark. The omitted types of care include nursing homes, home care, assistive technology for disabeled persons and the like, which may be a considerable omission for the elderly age groups in particular.

The analytical approach is that of a static model that assumes that anything other than age remains unchanged during the lifetime, including the cost and effectiveness of medical technology, and incidence, prevalence and severity of diseases. This assumption is clearly unrealistic. However, the great strength of the analytical approach is that these uncertain and complicated relations are avoided and only the consequences of older age are being modeled. However, it may be possible to incorporate more sophisticated assumptions by developing the modeling framework to account for this. A Canadian study has developed a Markow model to encompass such future changes.[18]

Another way of analyzing technological changes over time could be to employ the current model framework using longitudinal data. In this way it would be possible to compare the lifetime health care costs for a range of years, to observe trends over time. In such cases, it may be reasonable to formulate different scenarios in order to disentangle, for example, the effect of increasing life expectancy from that of technological developments.

In this study we made comparison between men and women. It might also be relevant to conduct the analysis stratified by different social groups e.g. by educational level, social/labor market position and/or groups with particular diseases or lifestyle behavior.

## 5. Conclusion

This study presents cost data for the general population that can be used to inform general economic models assessing the impact of aging populations and comparative models that seek to assess the efficiency and cost-effectiveness of new technologies. Future studies that address similar indicators for disease-specific populations will be valuable.

## Conflict of Interest Declaration

This study was supported by grants from the National Strategic Research Council (grant no. 2104-06-0027 and 2142-08-0017).
